# The circROBO1/KLF5/FUS feedback loop regulates the liver metastasis of breast cancer by inhibiting the selective autophagy of afadin

**DOI:** 10.1186/s12943-022-01498-9

**Published:** 2022-01-24

**Authors:** Zehao Wang, Lu Yang, Peng Wu, Xing Li, Yuhui Tang, Xueqi Ou, Yue Zhang, Xiangsheng Xiao, Jin Wang, Hailin Tang

**Affiliations:** 1 Department of Breast Oncology, Sun Yat-Sen University Cancer Center, State Key Laboratory of Oncology in South China, Guangzhou, China; 2 Department of Radiotherapy, Cancer Center, Guangdong Provincial People’s Hospital, Guangdong Academy of Medical Sciences, Guangzhou, China

**Keywords:** circROBO1, FUS, KLF5, miR-217, BC, Metastasis, Autophagy, Afadin, BECN1

## Abstract

**Background:**

Metastasis causes the majority of cancer-related deaths worldwide. Increasing studies have revealed that circRNAs are associated with the carcinogenesis and metastasis of many cancers. Nevertheless, the biological mechanisms of circRNAs in breast cancer (BC) liver metastasis remain extremely ambiguous.

**Methods:**

In this study, we identified circROBO1 from three pairs of primary BC and metastatic liver sites by RNA sequencing. FISH assays and RT-qPCR were conducted to validate the existence and expression of circROBO1. The oncogenic role of circROBO1 was demonstrated both in vitro and in vivo. Western blot, ChIP, RIP, RNA pull-down, and dual-luciferase reporter assays were used to confirm the interaction of the feedback loop among circROBO1, miR-217-5p, KLF5, and FUS. Meanwhile, the regulation of selective autophagy was investigated by immunofluorescence, CoIP, and western blot.

**Results:**

In this study, upregulated expression of circROBO1 was found in BC-derived liver metastases and was correlated with poor prognosis. Knockdown of circROBO1 strikingly inhibited the proliferation, migration, and invasion of BC cells, whereas overexpression of circROBO1 showed the opposite effects. Moreover, overexpression of circROBO1 promoted tumor growth and liver metastasis in vivo. Further research revealed that circROBO1 could upregulate KLF5 by sponging miR-217-5p, allowing KLF5 to activate the transcription of FUS, which would promote the back splicing of circROBO1. Therefore, a positive feedback loop comprising circROBO1/KLF5/FUS was formed. More importantly, we found that circROBO1 inhibited selective autophagy of afadin by upregulating KLF5.

**Conclusions:**

Our results demonstrated that circROBO1 facilitates the carcinogenesis and liver metastasis of BC through the circROBO1/KLF5/FUS feedback loop, which inhibits the selective autophagy of afadin by suppressing the transcription of BECN1. Therefore, circROBO1 could be used not only as a potential prognostic marker but also as a therapeutic target in BC.

**Supplementary Information:**

The online version contains supplementary material available at 10.1186/s12943-022-01498-9.

## Background

As the most prevalent type of cancer in women, breast cancer (BC) is the leading cause of cancer-related mortality among women worldwide. According to a recent report, the survival rate at 5 years for primary BC is almost 99%. However, approximately one-third of patients with BC present distant nonnodal metastasis, which decreases their 5-year survival rate to 23% [1]. BC mostly metastasizes to the lungs, bone, liver, and brain via the circulation, and the liver is the third most common organ site for BC metastasis. Fifty percent of BC patients with metastasis develop liver metastasis, and 5-12% of patients with BC develop liver metastasis as the primary site of recurrence [2]. If liver metastasis is left untreated, the patients’ survival time ranges from approximately 4 to 8 months [3]. However, current treatments for BC-derived liver metastasis are primarily based on systemic hormones and/or chemotherapy [4, 5], which only might extend the survival of patients to approximately 18-24 months [6]. Nevertheless, little is known about the concrete mechanism by which the invasive capability of cancer cells is enhanced in BC progression, which might be a promising target for precise antimetastatic treatment.

The majority of the transcriptome comprises noncoding RNAs, while 2% of the genome can be transcribed into messenger RNAs only [7]. Among the noncoding RNAs, circular RNAs (circRNAs) have a unique circular structure and were once thought to be “byproducts” of transcription. However, circRNAs have been shown to be widespread in cells and exhibit abundant biological regulatory functions. Compared with linear mRNAs, circRNAs have no traditional RNA structures, such as a 3′ polyadenylated tail or 5′ cap, which makes them more stable and evolutionarily conservative [8, 9]. As a popular research field, circRNAs have attracted much attention from researchers due to their ability to sponge miRNAs, bind and interact with proteins, and translate a novel protein to act like mRNAs. According to recent research, aberrant expression of circRNAs has been identified in a variety of human cancers [10–13]. All these findings revealed that circRNAs play a significant role in cancer progression and are extremely likely to be biomarkers of prognosis and new therapeutic targets in treatment. However, the biological function of circRNAs in the initiation of liver metastasis in BC has not yet been elucidated.

In our present study, we identified by RNA sequencing that the novel circRNA hsa_circ_0124696 (circROBO1) was upregulated in BC-derived liver metastases and correlated with poor survival. CircROBO1 promoted BC proliferation and metastasis in vivo and in vitro by increasing KLF5 expression, while FUS promoted the back splicing of circROBO1 and was activated transcriptionally by KLF5, resulting in a positive feedback loop. Moreover, circROBO1 could inhibit selective autophagy of afadin, a key biomarker of BC metastasis to the liver, via upregulating KLF5. Therefore, our research revealed for the first time the inhibition of selective autophagy of afadin driven by a positive feedback loop comprising circROBO1/KLF5/FUS, which promoted BC-derived liver metastasis progression and provided novel prognostic biomarkers and antimetastatic therapeutic targets for BC patients in clinical practice.

## Results

### CircROBO1 correlates with BC liver metastasis

**Fig. 1:**
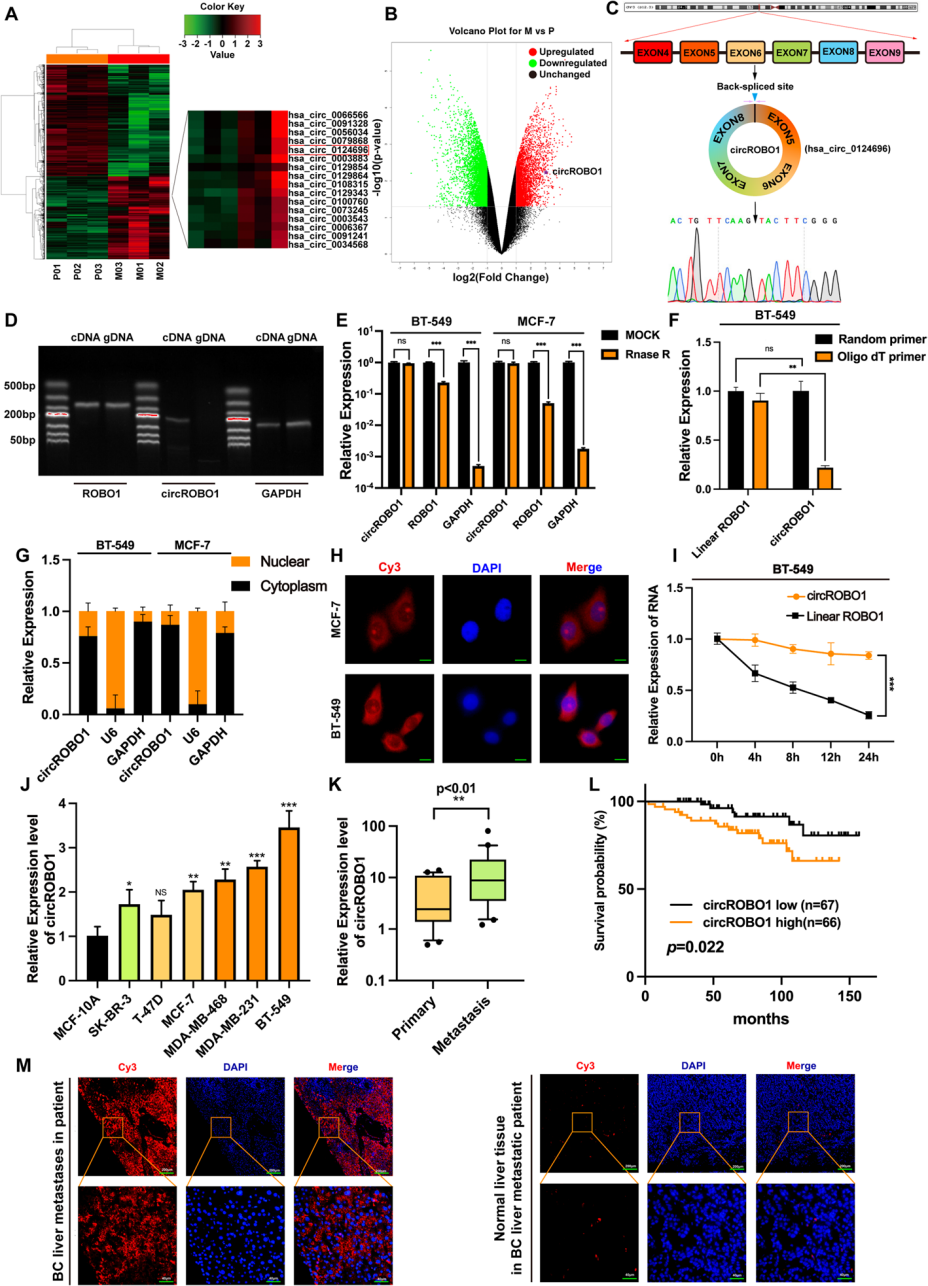
circROBO1 is validated and characterized in BC cells. **a** The cluster heat map showed that circROBO1 was in the upregulated expressed circRNAs in 3 pairs of human BC primary tissues and liver metastatic tissues. The red and green strips indicate upregulated and downregulated circRNAs. **b** The volcano plot showed that circROBO1 was one of the upregulated expressed circRNAs. **c** Schematic illustration of circROBO1 formation by the circulation of exon5 to exon8 in ROBO1 genes. The back-spliced junction sequences of circROBO1 were validated by Sanger sequencing. **d** PCR was conducted to detect the existence of circROBO1 and ROBO1 from gDNA and cDNA in BC cells respectively. Divergent primers amplified circROBO1 in cDNA rather than in gDNA while GAPDH was used as internal reference. **e** RT-qPCR was performed to determine the abundances of circROBO1 and linear ROBO1 mRNA in BC cells treated with Rnase R. **f** Oligo dT primers and random hexamers were used for the reverse transcription. The relative RNA levels were normalized by the value of random hexamer primers. **g** Nuclear-cytoplasmic fraction assay indicated that circROBO1 was localized in the cytoplasm of BC cells mostly. GAPDH was used as a cytoplasmic control and U6 was considered as a nuclear control. **h** The localization of circROBO1 was observed in BC cells by FISH assays (Scale bar=20μm). **I** The expression of circROBO1 and ROBO1 of BC cells were quantified by RT-qPCR after treatment for 4h, 8h, 12h, 24h. **j** RT-qPCR was performed to detect the expression of circROBO1 in different BC cells, normalized by GAPDH. **k** The expression of circROBO1 in paired BC primary tissues and liver metastatic tissues were analyzed by RT-qPCR and paired Student’s test, normalized by GAPDH (*n*=20)**. l** Kaplan-Meier survival analysis of BC patients according to the expression level of circROBO1 using log-rank test (*n*=133). **m** RNA FISH assays in human BC liver metastatic specimen showing significant circROBO1 overexpression. The data are showed as the mean ± SD, NS (no significance) *P < 0.05 **P < 0.01, ***P < 0.001 and all above experiments have been repeated for three times

To identify the differentially expressed circRNAs in liver metastases derived from BC, we performed high-throughput RNA-seq in 3 pairs of BC primary tumors and matched liver metastases. When we set the filter criteria to fold-change ≥ 2 and *P* value <0.01, we found 4735 differentially expressed circRNAs, among which 1941 were upregulated and 2794 were downregulated in liver metastatic tissues (Fig. 1a-b). Among the upregulated circRNAs, circROBO1 (hsa_circ_0124696), which consists of four exons (exon 5, exon 6, exon 7 and exon 8), was our main focus, and its back spliced junction site of exon 8 and exon 5 was verified by Sanger sequencing in BT-549 cells (Fig. 1c). To demonstrate that circROBO1 is back-spliced instead of trans-spliced, we designed divergent and convergent primers to amplify the circROBO1 transcript and linear ROBO1 transcript, respectively. The PCR results showed that circROBO1 was detected in cDNA using divergent primers only, while linear ROBO1 transcripts were detected in both cDNA and gDNA using convergent primers, which concluded that circROBO1 was the back-spliced transcript (Fig. 1d). Next, we found that circROBO1 had more resistance to degradation of Rnase R than did linear ROBO1 transcripts (Fig. 1e). Moreover, oligo dT primers and random hexamers were used for RNA reverse transcription. The results showed that linear ROBO1 transcripts were identified in both random hexamer and oligo dT primers, whereas circROBO1 was almost undetectable using oligo dT primers, which is consistent with the characteristics of circRNA owing to their lack of a poly-A tails (Fig. 1f). To further evaluate the stability of circROBO1, an actinomycin D test was conducted to identify the different metabolic half-lives of circROBO1 and linear ROBO1 transcripts. The results showed that circROBO1 was more stable and had a distinctly longer half-life than did linear ROBO1 transcripts after treatment with actinomycin D (Fig. 1i). The above results suggested that circROBO1 was a back-spliced circRNA. Then, we extracted the cytoplasmic and nuclear fractions of BT-549 cells and MCF-7 cells and performed RT-qPCR to identify the subcellular localization of circROBO1. The results showed that circROBO1 was mostly located in the cytoplasm rather than the nucleus (Fig. 1g). FISH assays also confirmed the same results (Fig. 1h).

To identify the expression of circROBO1 in BC cell lines and liver metastatic samples, we performed RT-qPCR and found that circROBO1 was significantly upregulated in BC cells compared with MCF-10A cells (Fig. 1j). Moreover, the results of RT-qPCR in patient-matched samples showed that circROBO1 was upregulated in BC-derived liver metastases compared to BC primary samples (Fig. 1k). Meanwhile, Kaplan–Meier analysis indicated that BC patients with increased expression of circROBO1 had shorter overall survival (Fig. 1l). Next, RNA FISH assays were performed and showed that the expression of circROBO1 was significantly upregulated in BC-derived liver metastases compared with adjacent normal liver tissues (Fig. 1m). All these findings suggest that circROBO1 plays an important role in BC liver metastasis.

### CircROBO1 is essential for BC proliferation and metastasis in vitro

**Fig. 2:**
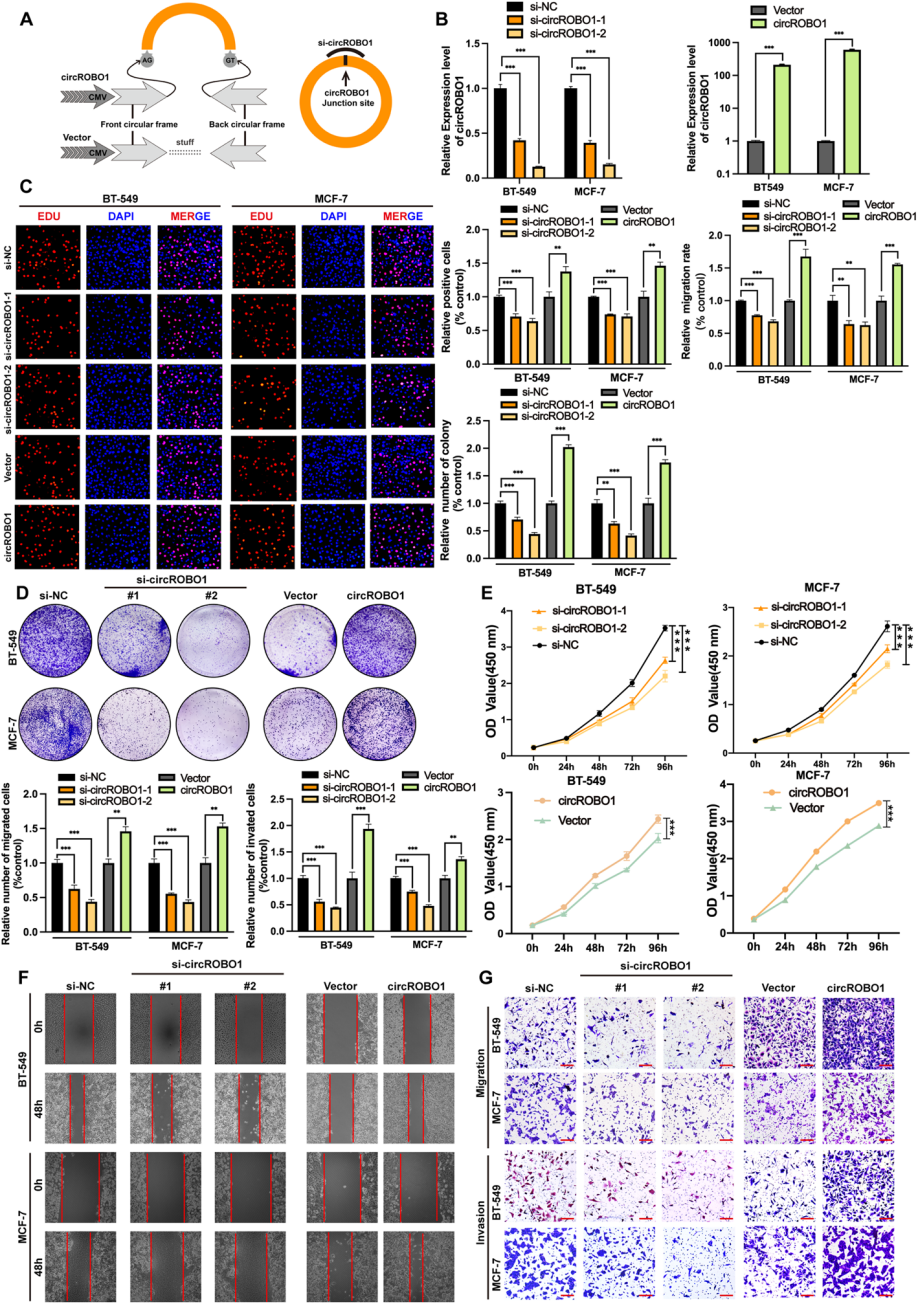
Knockdown of circROBO1 inhibits proliferation, migration, and invasion of BC cells and overexpression of circROBO1 elicits the opposite effects. **a** The schematic illustration of circROBO1 overexpression vector and knockdown of circROBO1 via siRNAs. **b** RT-qPCR was performed to measure the efficiency of overexpression and knockdown of circROBO. **c** Edu assays of BC cells was conducted to evaluate proliferation (Scale bar=50μm). **d** Colony formation was performed to evaluate the cell proliferation ability. **e** CCK8 assays were used for analyzing the growth curves of cells. **f** and **g** Wound healing assays and Transwell migration, invasion assays (Scale bar=100μm) were performed to measure the migration and invasion abilities in BC cells. The data are showed as the mean ± SD, *P < 0.05 **P < 0.01, ***P < 0.001 and all above experiments have been repeated for three times

To investigate the potential function of circROBO1 in BC, we designed two small interfering RNAs (siRNAs) and constructed a circROBO1 overexpression plasmid (Fig. 2a). Then, RT-qPCR was performed to verify that circROBO1 was significantly downregulated or overexpressed in BC cells after transfection with siRNAs or overexpression plasmids, respectively (Fig. 2b). Si-circROBO-2 was the most efficient siRNA in silencing circROBO1, therefore, it was selected for subsequent experiments. Nevertheless, the expression of the linear ROBO1 transcript had no significant change after overexpression or knockdown of circROBO1 (Fig. S 1a). Next, we performed EdU, CCK-8, and colony formation assays to detect the viability of BC cells. The results showed that the proliferation ability of BC cells was notably increased after overexpression of circROBO1 and significantly suppressed after knockdown of circROBO1 (Fig. 2c-e). Moreover, we performed Transwell and wound healing assays and found that overexpression of circROBO1 upregulated the migration and invasion of BC cells, while knockdown of circROBO1 exerted the opposite effects (Fig. 2f-g). All the results suggest that circROBO1 acts as an oncogene in BC cells.

### CircROBO1 accelerates the growth of xenograft tumors and liver metastasis in vivo

**Fig. 3:**
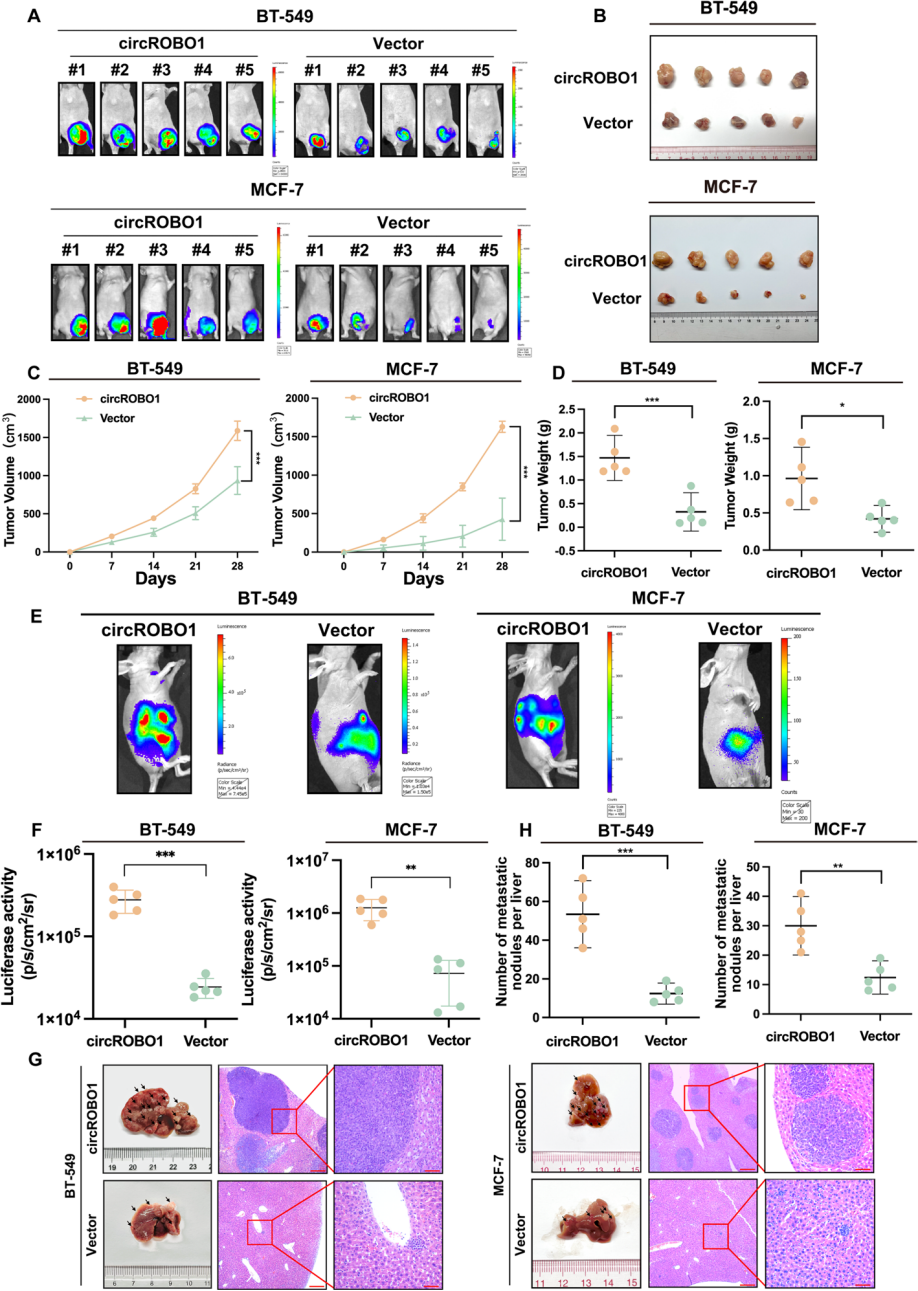
circROBO1 promotes carcinogenesis and liver metastasis of BC cells. **a** Subcutaneous injection model to verify the carcinogenetic potential of circROBO1 overexpression group and control group by IVIS. **b** The images of xenograft tumor in circROBO1 overexpression group and control group were displayed (*n*=5). **c** The volumes of tumor were measured once a week and the growth curves of each group were drawn. **d** The weight of tumors were measured and analyzed. **e** and **f** In vivo inferior hemispleen implantation models to analyze the liver metastasis potential of circROBO1 by IVIS. **g** and **h** The metastatic sites vividly increased in circROBO1 overexpression group. Metastatic nodules of livers were labeled by arrows and H&E staining of the livers and tumors were showed. The data are showed as the mean ± SD, *P < 0.05 **P < 0.01, ***P < 0.001

To evaluate whether circROBO1 affected tumor growth in vivo, human BC xenograft models were established with BT-549 cells and MCF-7 cells stably overexpressing circROBO1, and their corresponding control cells were injected subcutaneously into female BALB/C nude mice. The results revealed that the weight and volume of tumors in the circROBO1 overexpression group were significantly higher than those in the control group for both BT-549 cells and MCF-7 cells (Fig. 3a-d). To further evaluate the function of circROBO1 in BC liver metastasis, we injected BT-549 cells, MCF-7 cells with stable circROBO1 overexpression, and their corresponding control cells into the inferior hemispleen to mediate liver metastasis. The results showed that the number of liver metastatic nodules in the circROBO1 overexpression group was strikingly higher than that in the control group (Fig. 3e-h). All the results were consistent with the in vitro results, suggesting that circROBO1 could promote BC tumorigenesis and liver metastasis.

### FUS modulated the back splicing of circROBO1

**Fig. 4:**
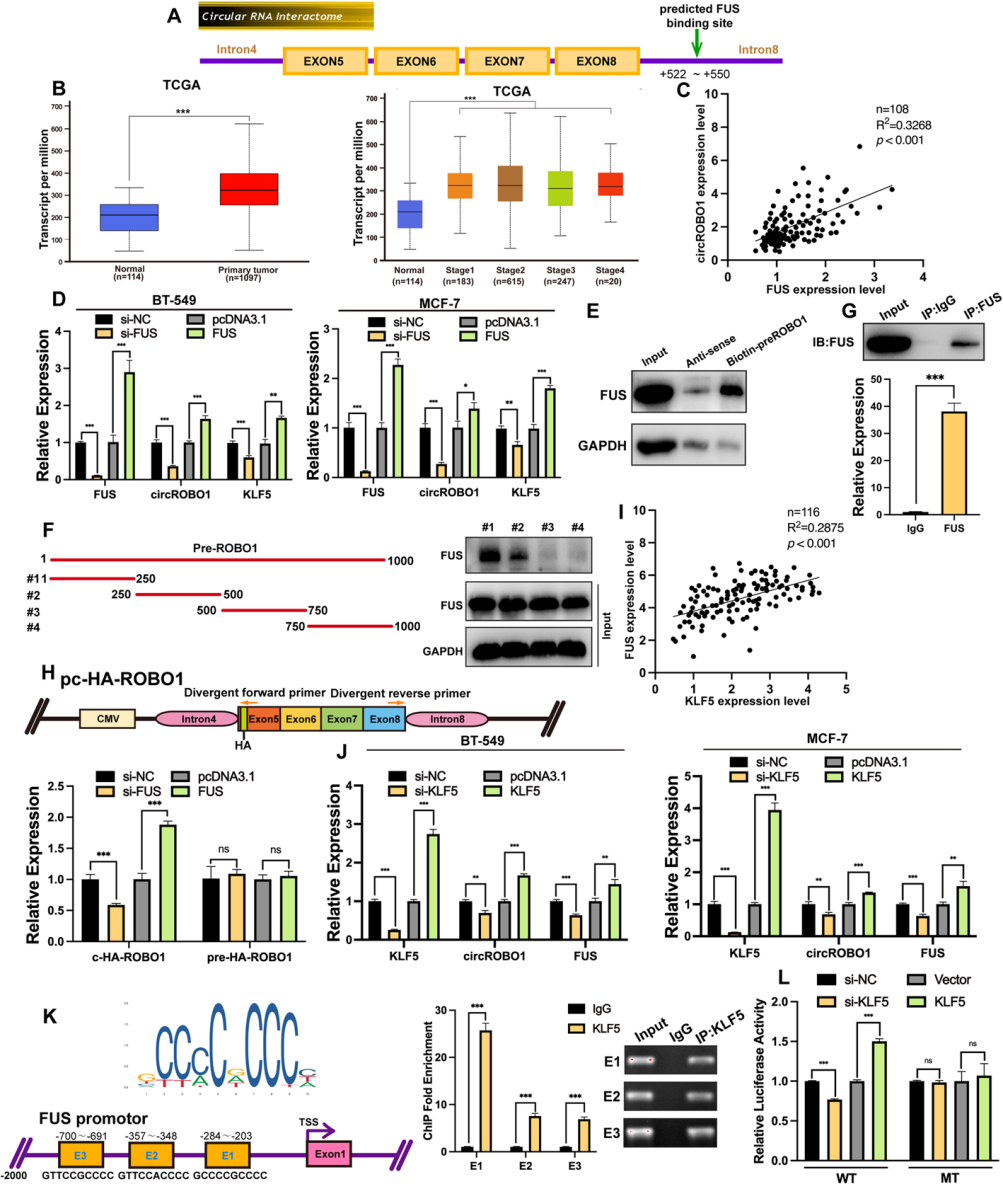
FUS promotes biogenesis of circROBO1 and KLF5 enhances transcription of FUS. **a** According to prediction of CircInteractome, an online database (https://circinteractome.nia.nih.gov/), the flanking intron downstream of circROBO1 harbored binding sites of FUS. **b** FUS was upregulated in BC according to the TCGA database and upregulated in I-IV stage of BC. **c** The Pearson correlation analysis showed that the mRNA expression of circROBO1 had a positive correlation with FUS in human BC tissues. **d** Knockdown of FUS or overexpression of FUS significantly decreased or increased the expression of circROBO1 in BC cells. **e** The results of RNA pull-down assays analyzed by western blot. **f** Schematic illustration of truncated pre-ROBO1 biotin-labeled probes used in RNA pull-down assays and the results of RNA pull-down assays were analyzed by western blot. **g** RIP assay was conducted using BT-549 lysates and FUS antibodies. Enrichment of pre-ROBO1 was analyzed by RT-qPCR. **h** Schematic diagram illustrated the structure of back splicing formation verification vector pc-HA-ROBO1 by inserting a HA tag in the 5’ side of exon 5 of circROBO1 to discriminate the internal circROBO1 with its downstream and upstream flanking 1000 nucleotide introns into the pcDNA3.1+ vector. The results of RT-qPCR by divergent primers crossed HA tag were displayed. **i** The Pearson correlation analysis showed that the mRNA expression of KLF5 had a positive correlation with FUS in human BC tissues. **j** Knockdown or overexpression of KLF5 downregulated or upregulated mRNA level of FUS analyzed by RT-qPCR. **k** and **l** JASPAR database indicated the potential KLF5 binding sites on the promotors of FUS. ChIP assays were conducted to verify the enrichment of the potential binding sites by KLF5 antibody in BT-549 cells. The ChIP results were analyzed by qPCR and nucleic acid electrophoresis. The dual-luciferase reporter assays were performed to validate the interaction between KLF5 and the first binding site (E1). The data are showed as the mean ± SD, *P < 0.05 **P < 0.01, ***P < 0.001 and all above experiments have been repeated for three times

According to the CircInteractome database (https://circinteractome.nia.nih.gov/), the 3′ start flanking intron of circROBO1 has a potential FUS binding region (Fig. 4a). First, the expression of FUS was higher in BC samples than in normal tissues, while the expression of FUS in TNM stage I-IV BRCA tissues was upregulated compared to that in normal tissues, which indicated that FUS is highly expressed in BC and has the potential to be an oncogene in BC (Fig. 4b). Then, RT-qPCR was performed in 108 BC primary tissues to identify that circROBO1 was positively correlated with FUS (Fig. 4c). Next, RT-qPCR showed that the expression of circROBO1 was positively related to the expression level of FUS in BT-549 and MCF-7 cells when FUS was ectopically overexpressed and knocked down (Fig. 4d). According to previous reports, FUS is a modulator that promotes the biogenesis of back splicing of circRNAs [14]. Therefore, we hypothesized that FUS aids in the process of back splicing of ROBO1 pre-mRNA (pre-ROBO1) to promote the formation of circROBO1 by binding to its 3′ start flanking intron. To further investigate this hypothesis, we designed a biotin-labeled pre-ROBO1 probe that contains 1000 nucleotides downstream of exon 8 of ROBO1. Then, we performed an RNA pull-down assay and found that the pre-ROBO1 probe successfully captured the FUS protein compared to the antisense probe (Fig. 4e). Nevertheless, to further pinpoint the binding region of FUS, we designed four truncated biotin-labeled probes based on the pre-ROBO1 probe and extracted the lysates of BT-549 cells to perform RNA pulldown assays. The results showed that the first truncated probe of pre-ROBO1, was mostly enriched with FUS (Fig. 4f). Then, an RIP assay was used to confirm the interaction between FUS and pre-ROBO1 (Fig. 4g). To further show that FUS plays a direct role in the back-splicing process of pre-ROBO1, we constructed the pc-HA-ROBO1 vector and verified the efficiency of back splicing of circROBO1. After cotransfection with a FUS overexpression vector or FUS-targeted siRNA, RT-qPCR was performed to show that the back splicing biogenesis of ectopically expressed circROBO1 was correlated with the expression level of FUS, while the expression of pre-ROBO1 was not influenced by FUS in HEK293T cells (Fig. 4h). All results suggest that FUS could bind with the 3′ start flanking intron region of pre-ROBO1 to promote the back splicing of circROBO1.

### CircROBO1 binds to miR-217-5p directly and suppresses miR-217-5p activity

**Fig. 5:**
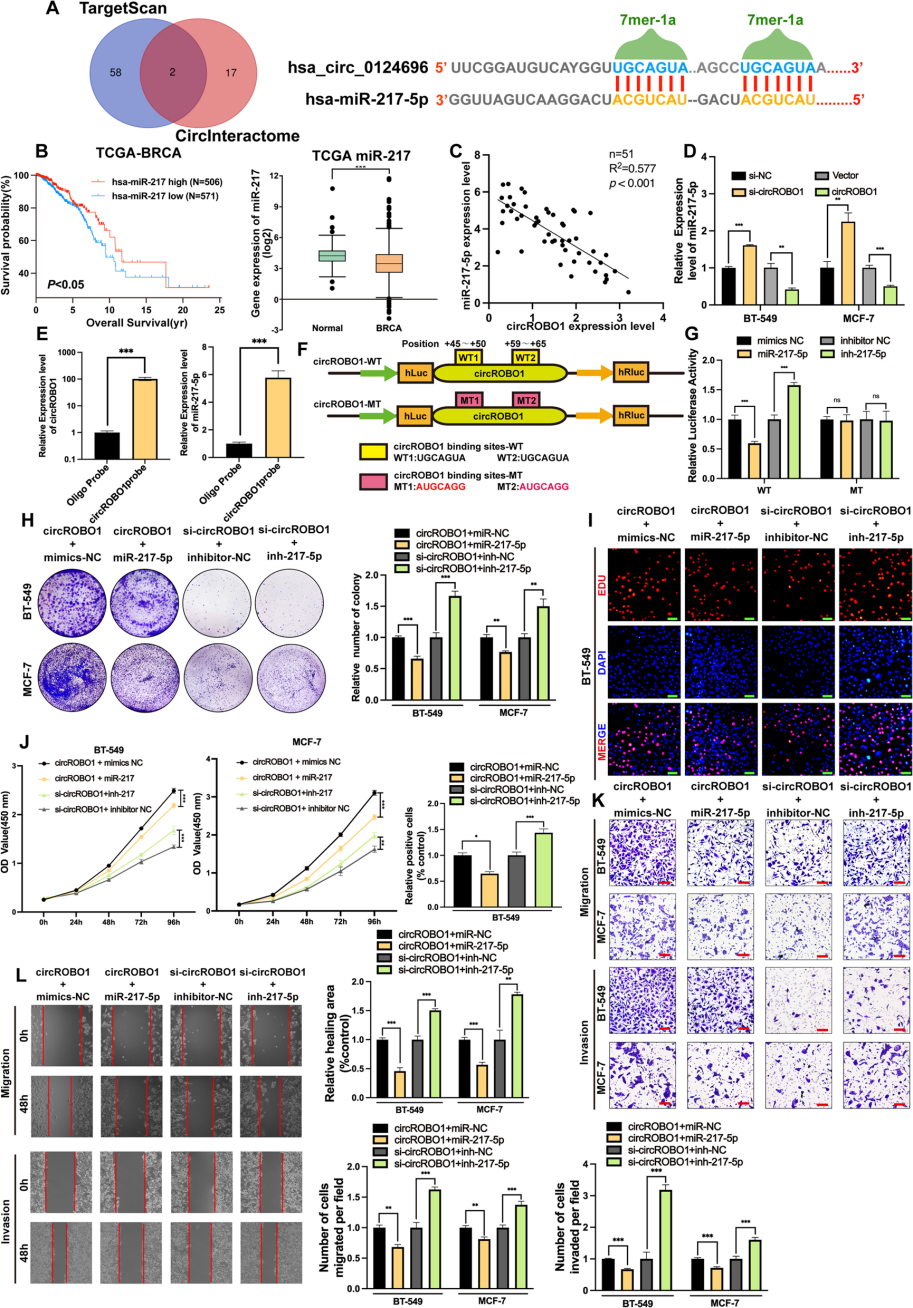
CircROBO1 functions as a sponge of miR-217-5p. **a** Venn diagram showed the intersection of TargetScan and Circinteractome in potential miRNA binding sites of circROBO1. The two potential binding sites of miR-217-5p in circROBO1 was displayed. **b** According to the TCGA database, miR-217 was downregulated in BC and indicated a favorable prognosis in BC. **c** RT-qPCR was performed in 51 pairs of BC tissues which validated there existed a negative correlation between miR-217-5p and circROBO1. **d** Knockdown or overexpression of circROBO1 upregulated or downregulated miR-217-5p by RT-qPCR. **e** RNA pull-down assays with a biotin-labeled probe of junction site sequences of circROBO1 were performed in BT-549 to validate the interaction between circROBO1 and miR-217-5p and the enrichment were detected by RT-qPCR to test the enrichment of miR-217-5p. **f** Schematic illustration displayed the dual-luciferase report vectors with wild-type (WT) and mutant-type (MT) miR-217-5p binding sites. **g** The relative activities of luciferase were detected after co-transfection of circROBO1-WT or circROBO1-MT and miR-217-5p mimics or inhibitors with control respectively in 293T cells. **h-j** The cell viability of BC cells were detected after co-transfection with indicated siRNAs, vectors, miRNAs or inhibitors by colony formation assays, Edu assays (Scale bar=50μm), and CCK8 assays respectively. **k** and **l** The migration and invasion abilities of BC cells co-transfected with indicated siRNAs, vectors, miRNAs or inhibitors were measured by transwell migration and invasion assays (Scale bar=100μm) and wound healing assays respectively. The data are showed as the mean ± SD, *P < 0.05 **P < 0.01, ***P < 0.001 and all above experiments have been repeated for three times

To further study the biological mechanism of circROBO1, we performed bioinformatic analysis to predict the potential binding miRNAs of circROBO1 in the CircInteractome (https://circinteractome.nia.nih.gov/) and TargetScan databases (http://www.targetscan.org). The results showed that miR-217-5p is the only miRNA that has two binding sites for circROBO1 in the intersection between the CircInteractome and TargetScan databases, which indicated that circROBO1 might act as a miRNA sponge in BC (Fig. 5a). According to the TCGA database, miR-217-5p was significantly downregulated in BC compared to normal tissue in 1165 samples, while BC patients with higher miR-217-5p expression had better overall survival (Fig. 5b). These results indicated that miR-217-5p might act as a tumor suppressor. Next, we performed RT-qPCR in 51 pairs breast cancer tissues to find that there existed a negative correlation between circROBO1 and miR-217-5p (Fig. 5c). Then, RT-qPCR was performed and showed that knockdown and overexpression of circROBO1 significantly increased and decreased the expression of miR-217-5p in BC cells, respectively (Fig. 5d). After that, we designed a biotin-labeled junction site probe of circROBO1 and performed an RNA pulldown assay, which showed that miR-217-5p was abundantly pulled down by the probe compared to that in the control group (Fig. 5e). To further confirm the interaction between circROBO1 and miR-217-5p, we constructed wild-type and mutant-type dual-luciferase reporter plasmids containing binding sites between miR-217-5p and circROBO1. The results showed that miR-217-5p mimics significantly suppressed the luciferase activity of the wild-type group, while miR-217-5p inhibitors promoted luciferase activity, whereas the mutant group had no detectable change after either treatment (Fig. 5f-g), which indicated that circROBO1 could bind with directly miR-217-5p and act as a sponge of miR-217-5p. To further research the biological interaction between circROBO1 and miR-217-5p in BC cells, we carried out rescue experiments by cotransfecting miR-217-5p mimics or miR-217-5p inhibitors with circROBO1 overexpression vector or si-circROBO1, respectively, into BT-549 cells and MCF-7 cells. The results of colony formation assays, CCK-8, EdU, Transwell, and wound healing assays indicated that ectopic overexpression of miR-217-5p remitted the proliferation, migration and invasion effects induced by upregulation of circROBO1 in BT-549 cells and MCF-7 cells, while miR-217-5p inhibitors reversed the suppressive effects of proliferation, migration and invasion caused by knockdown of circROBO1 (Fig. 5h-l). In summary, circROBO1 could act as a ceRNA to reverse the inhibitory role of miR-217-5p to promote the initialization and progression of BC.

### KLF5 is a direct target of miR-217-5p and activates the mTOR-PI3K-AKT pathway through the circROBO1/miR-217-5p/KLF5 axis

**Fig. 6:**
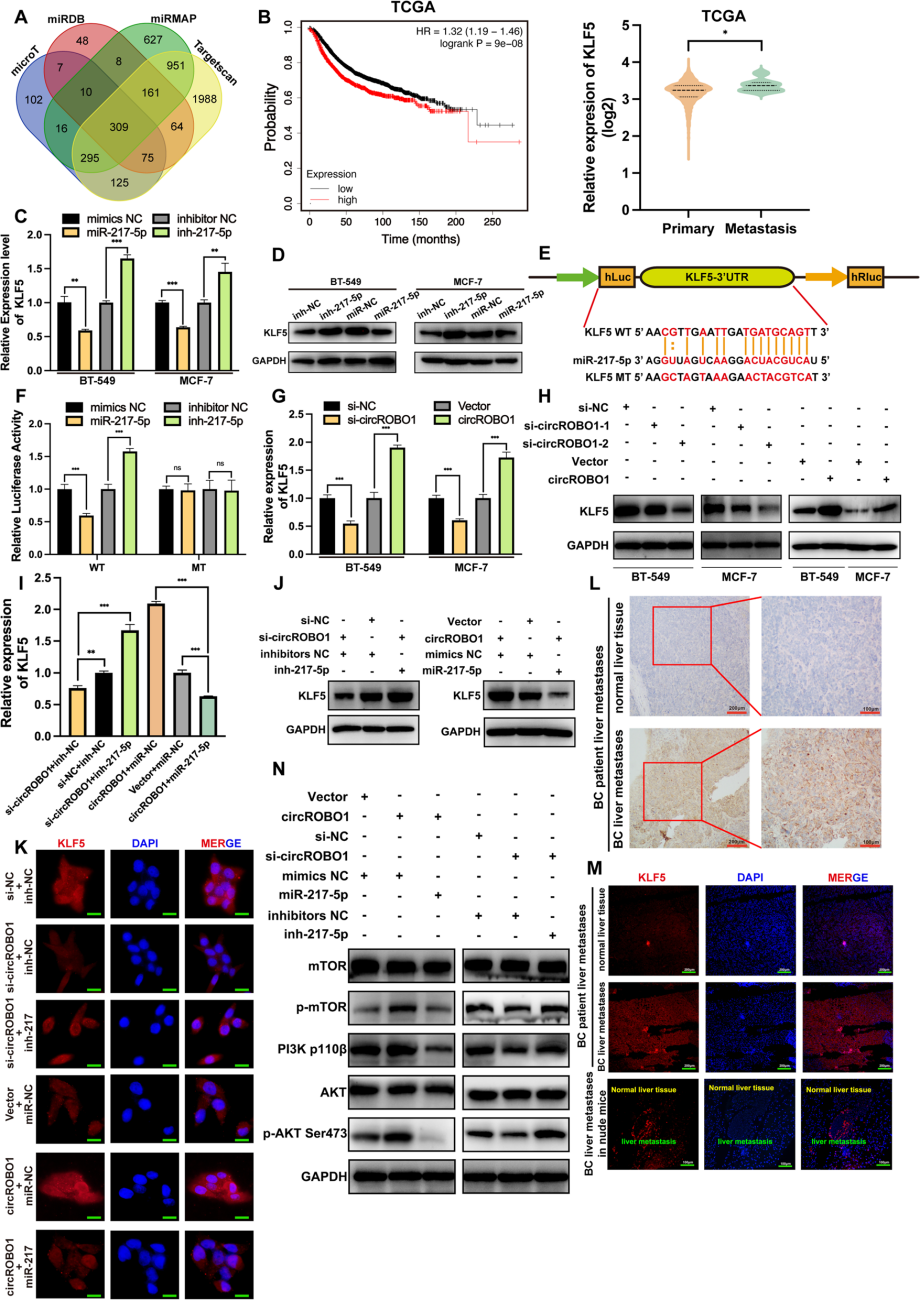
KLF5 is directly targeted by miR-217-5p. **a** Venn diagram showed the potential target genes of miR-217-5p predicted by miR-217-5p targeted genes among microT, miRDB, miRMAP, and Targetscan. **b** According to the TCGA database, KLF5 was upregulated in BC patients with metastasis and predicted a poor prognosis. **c** and **d** The relative expression of KLF5 were analyzed by RT-qPCR and western blot after transfection with miR-217-5p mimics and inhibitors. **e** Schematic illustration of KLF5 3’UTR wild type (WT) and mutant type (MT) were displayed. **f** The dual-luciferase assays indicated that miR-217-5p directly bind to the 3’UTR of KLF5 and inhibit the activity of luciferase. **g** and **h** Knockdown or overexpression circROBO1 decreased or increased the mRNA and protein level of KLF5 analyzed by RT-qPCR and western blot. **i**-**k** The mRNA and protein expression level of KLF5 were detected by RT-qPCR, western blot, and immunofluorescence (IF) (Scale bar=20μm) after co-transfection with indicated vectors, siRNAs, mimics or inhibitors. **l** and **m** IF and immunohistochemistry (IHC) were performed in human liver metastatic specimens. **n** circROBO1 activated the pathway of mTOR, phosphorylated mTOR, PI3K 110β, AKT, and phosphorylated AKT verified by western blot after co-transfection with indicated vectors, siRNAs, mimics or inhibitors. The data are showed as the mean ± SD, *P < 0.05 **P < 0.01, ***P < 0.001 and all above experiments have been repeated for three times

To further study the potential mechanism involved in this activity, we conducted bioinformatics analysis with the TargetScan (http://www.targetscan.org), microT (https://bio.tools/DIANA-microT), miRDB (http://mirdb.org) and miRMap (https://mirmap.ezlab.org) databases. The data indicated that KLF5 contains conserved binding sites for miR-217-5p in the intersection of the results of these four databases (Fig. 6a). Then, analysis of the TCGA data from the online KMPlotter database (http://kmplot.com/analysis/) revealed that higher expression of KLF5 predicted poor relapse-free survival in BC. Additionally, the data from the TCGA showed that KLF5 expression was higher in BC patients with metastasis than in BC patients without metastasis (Fig. 6b). Meanwhile, we found that expression of KLF5 was significantly upregulated in triple negative breast cancer compared with luminal and HER2+ subtypes in TCGA (Fig. S 1d). Moreover, we performed immunohistochemistry (IHC) and immunofluorescence (IF) of BC-derived liver metastasis samples from humans, and the results showed that the expression of KLF5 was significantly higher in liver metastatic samples than in normal liver tissues by both IHC and IF (Fig. 6l-m). All the above results suggested that KLF5 was positively correlated with BC liver metastasis and predicted poor survival in BC. Next, RT-qPCR and western blot were performed and showed that KLF5 was significantly downregulated at both the mRNA and protein levels in BT-549 and MCF-7 cells transfected with miR-217-5p mimics, whereas cells transfected with miR-217-5p inhibitors showed markedly opposite effects (Fig. 6c-d). To further verify the interaction between KLF5 and miR-217-5p, we performed a dual-luciferase reporter assay. We cloned the 3′-untranslated regions (3′UTRs) of KLF5 from gDNA into pmirGLO as wild-type group, while mutant-type group was established with mutations at the predicted binding sites for miR-217-5p. After cotransfection of these plasmids with miR-217-5p mimics or inhibitors, we found that luciferase activity was significantly inhibited by miR-217-5p mimics and promoted by inhibitors in the wild-type group. However, the luciferase activity showed almost no changes in the mutant-type group after mimic or inhibitor treatment (Fig. 6e-f). To further confirm the relationship between circROBO1 and KLF5, we overexpressed and knocked down circROBO1, and RT-qPCR western blot were conducted and showed that the mRNA and protein expression of KLF5 had a distinctly positive correlation with circROBO1 (Fig. 6g-h). Moreover, we transfected miR-217-5p mimics in the circROBO1 overexpressing group and miR-217-5p inhibitors in the circROBO1 knockdown group as rescue experiments and found that miR-217-5p mimics significantly reversed the upregulation of KLF5 in the circROBO1 overexpressing group, while miR-217-5p inhibitors markedly increased the expression of KLF5 in the circROBO1 knockdown group at the mRNA and protein levels (Fig. 6i-k). In summary, circROBO1 could upregulate the expression of KLF5 by sponging miR-217-5p to reverse the suppression of KLF5 by miR-217-5p. Moreover, we performed western blot to show that overexpression or knockdown of circROBO1 could increase or decrease, respectively, the protein expression levels of phosphorylated mTOR (p-mTOR), PI3K, and phosphorylated AKT (p-AKT), while cotransfection with miR-217-5p mimics or inhibitors markedly reversed the upregulated or downregulated effects, respectively (Fig. 6n). All the above results suggested that circROBO1 could activate the KLF5-mTOR-PI3K-AKT pathway to promote BC liver metastasis.

### KLF5 promoted the transcription of FUS and formed a positive feedback loop with circROBO1

Interestingly, when we ectopically overexpressed and knocked down KLF5, the expression of FUS and circROBO1 was significantly increased and decreased, respectively (Fig. 4j), whereas the expression of the linear ROBO1 transcript was unchanged (Fig. S 1b). This indicated that KLF5 could not function as a transcriptional activator of ROBO1. Then, RT-qPCR was performed in 116 primary BC tissues and indicated that KLF5 was positively correlated with FUS at the mRNA level (Fig. 4i). Next, three potential binding sites of KLF5 were predicted in the promoter of FUS by JASPAR (http://jaspar.genereg.net/). Therefore, we speculated that KLF5 upregulated the expression of circROBO1 by increasing the expression level of FUS. To confirm our hypothesis, we performed a ChIP assay and found that E1 (-284~-203) was the most efficient of the three predicted binding sites (E1, E2, E3) in enriching KLF5 protein expression (Fig. 4k). To further validate the interaction between KLF5 and the E1 region, a luciferase reporter assay was performed. We cotransfected the KLF5 overexpression plasmid or KLF5 siRNA with the wild-type luciferase reporter plasmid containing 2000 bps of the FUS promoter or with a reporter plasmid with mutations in the E1 region of the FUS promoter. The results showed that the luciferase signals had a positive correlation with the expression of KLF5 in the wild-type group, whereas there was no correlation in the mutant-type group, which indicated that KLF5 could activate the transcription of FUS to upregulate the expression of circROBO1 (Fig. 4l). All the above results suggested that circROBO1/KLF5/FUS formed a positive feedback loop.

### CircROBO1 inhibited selective autophagy of afadin by downregulating BECN1

**Fig. 7:**
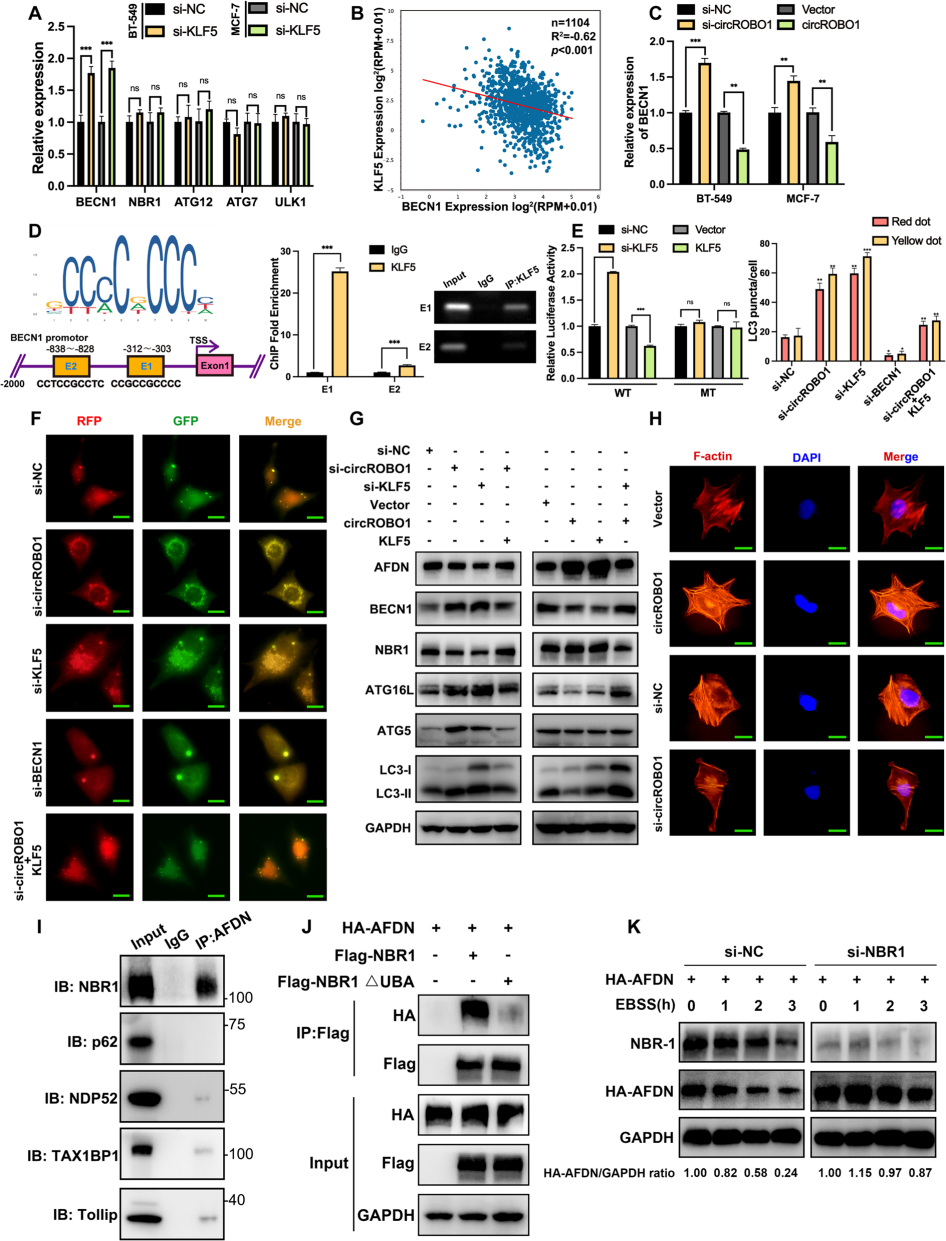
circROBO1 inhibits selective autophagy of afadin via KLF5. **a** RT-qPCR was performed to detect the mRNA change of genes associated with autophagy. **b** According to STARBASE, BECN1 was negatively correlated with KLF5. **c** After knockdown or overexpression of circROBO1, the change of BECN1 in mRNA level was analyzed by RT-qPCR. **d** Schematic diagram showed that two binding sites of KLF5 in BECN1 promotors were predicted by JASPAR. ChIP, qPCR and nucleic acid electrophoresis results indicated that the first one is the most enriched. **e** Dual-luciferase report assays were conducted to show that the luciferase activity was disappear in E1 mutant group. **f** Autophagosomes in mCherryGFP-LC3 labeled BT-549 cells after transiently transfected with siRNAs of circROBO1, KLF5, BECN1 respectively and rescued si-circROBO1 group with KLF5 overexpression vector (Scale bar=20μm). Quantification of the number of autolysosomes (red LC-3 puncta) and autophagosomes (yellow LC-3 puncta) per cell. The rescue group “si-circROBO1+KLF5” was compared to si-circROBO1 group and the others were compared to the NC control group. **g** The alteration of protein associated with autophagy and afadin was detected by western blot after transiently transfected with indicated siRNAs or vectors. **h** The F-actin was detected by IF in BT-549 cells transfected with siRNA or circROBO1 overexpression vector respectively (Scale bar=20μm). **i** After coimmunoprecipitation (CoIP) by antibody of afadin in BT-549 cell lysates, immunoblots with NBR1, p62, NDP52, TAX1BP1, Tollip were performed. **j** CoIP and immunoassay of lysates of 293T cells transfected with Flag-tagged wild-type NBR1 or mutant-type with UBA domain deletion, together with HA-tagged afadin, were performed. **k** After transfected with NBR1 siRNA for 12h, HA-tagged afadin was co-transfected for another 48h. Then EBSS was used to treat the cells for different hours and the results were determined by western blot

**Fig. 8:**
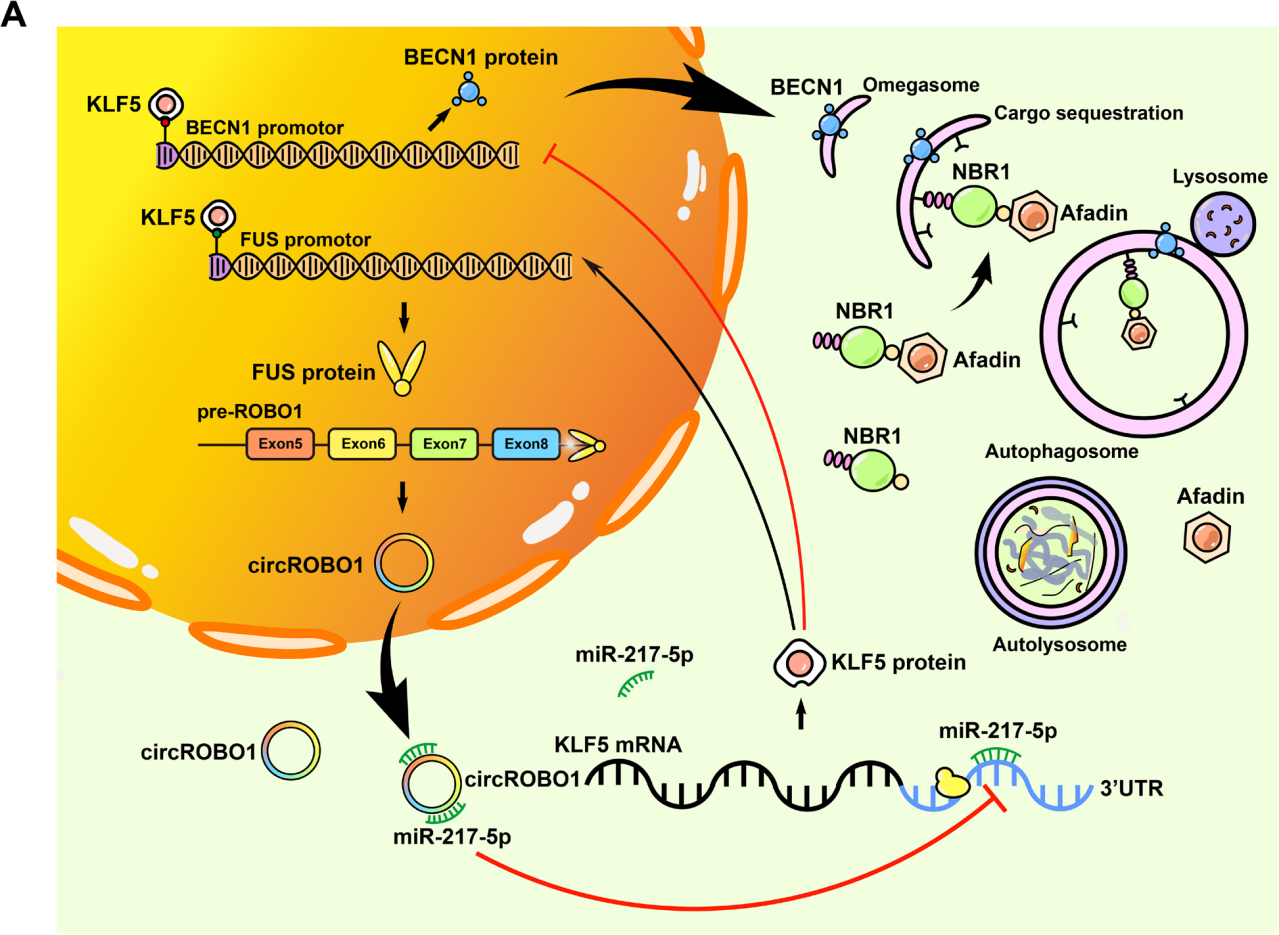
**a** Schematic diagram elucidates the mechanism of circROBO1/KLF5/FUS feedback loop which mediates inhibition of the selective autophagy of afadin

We performed RT-qPCR and found that among the target mRNAs involved in autophagy, BECN1 was the most upregulated after knockdown of KLF5 (Fig. 7a). Meanwhile, the results of starBase (http://starbase.sysu.edu.cn) analysis supported that the correlation between KLF5 and BECN1 was significantly negative (Fig. 7b). Moreover, the expression of BECN1 was downregulated in HER2+ and triple negative subtypes of BC compared to the normal group (Fig. S 1e). Then, RT-qPCR was conducted to show that BECN1 was increased after knockdown of circROBO1, which is consistent with the results of the KLF5 knockdown experiments (Fig. 7c). To further research the interaction between KLF5 and BECN1, we predicted two binding sites of KLF5 on the promoters of BECN1 via JASPAR (http://jaspar.genereg.net/). After that, we performed ChIP assays and found that KLF5 was significantly enriched in the predicted binding sites, with E1 (-312~-303) showing the most binding (Fig. 7d). Next, we cloned 2000 bp of the BECN1 promoter into the pEZX-FR01 plasmid; there was the wild-type group and mutant-type group in which the E1 region in the promoter was mutated. Dual-luciferase assays were conducted to reveal that the luciferase signals had a negative correlation with the expression level of KLF5 in the wild-type group, whereas this was not observed change in the mutant-type group (Fig. 7e), which indicated that KLF5 could inactivate the transcription of BECN1 to inhibit the process of autophagy in BC. To further validate the status of autophagy, we transfected the mCherry-EGFP-LC-3-BT-549 cells with circROBO1, KLF5 or BECN1 targeted siRNAs or with the KLF5 overexpression vector in circROBO1 knockdown group as the rescue group. The results showed that the knockdown of circROBO1 and KLF5 significantly increased the number of red and yellow puncta, while BECN1 knockdown significantly decreased the number of red and yellow puncta compared with that in the control group. Moreover, KLF5 overexpression reversed the upregulation of red and yellow dots caused by knockdown of circROBO1, which indicated that KLF5 plays an important role in the inhibition of autophagy activated by circROBO1 (Fig. 7f). Moreover, knockdown of circROBO1 or KLF5 increased the protein levels of LC3-B, ATG5, and ATG16L1, while cotransfection with the KLF5 overexpression vector in circROBO1 knockdown group significantly reversed this upregulation. Moreover, when circROBO1 or KLF5 was overexpressed or when circROBO1 overexpressed group  was cotransfected with si-KLF5, the effects were completely reversed (Fig. 7g). To further investigate the mechanism of the circROBO1/KLF5/FUS loop, IF was used, which find that F-actin was positively associated with the expression of circROBO1 (Fig. 7h). Recent research revealed that afadin, an F-actin-binding protein, plays an important role in BC liver metastasis, and a lack of afadin significantly diminished the colony-forming ability of BC cells and metastasis formation in the liver [15, 16]. Next, we found that the protein level of afadin was not only correlated with the expression of circROBO1 but also associated with the status of autophagy as assessed by western blot, whereas the mRNA level of afadin remained almost unchanged (Fig. S 1c), which made us consider that afadin might be degraded by selective autophagy at the protein level. Therefore, we conducted CoIP to screen the potential binding partners of afadin among p62, NBR1, NDP52, TAX1BP1 and Tollip, which are cargo receptors of the autophagy process (Fig. 7i). The results showed that afadin only interacted with NBR1, and the interaction between afadin and NBR1 did not occur if the UBA domain of NBR1 was disrupted, which indicated that NBR1 interacted with afadin via the UBA domain (Fig. 7j). We found by western blot that the inhibition of autophagy by the circROBO1/KLF5/FUS loop increased the protein level of NBR1, and we found that the degradation of afadin induced by EBSS was clearly reversed when NBR1 was knocked down (Fig. 7k). All the above results indicated that the circROBO1/KLF5/FUS positive feedback loop inhibited the selective autophagy of afadin by inhibiting the transcription of BECN1(Fig .8a).

## Methods

### Cell lines and culture conditions

Six human BC cell lines, BT-549, MDA-MB-231, MDA-MB-468, MCF-7, T-47D and SKBR-3; the normal breast epithelial cell line MCF-10A and the 293T cell line were purchased from ATCC. BT-549, MDA-MB-468, MDA-MB-231, T-47D, MCF-7, SKBR-3, and 293T cells were cultured in DMEM (Gibco, USA), while MCF-10A cells were cultured in DMEM/F-12 medium (Gibco, NY, USA). The cell media were supplemented with 50 U/ml penicillin and streptomycin (Gibco, NY, USA) and 10% fetal bovine serum (Gibco, NY, USA). All cells were cultured in a humidified incubator (Thermo Fisher Scientific, MA, USA) at 37 °C with 5% CO2.

### Patients and samples

All primary BC samples and liver metastasis samples were obtained from patients at the Sun Yat-Sen University Cancer Center (SYSUCC) in Guangzhou, China. All samples were instantly submerged in RNA later upon collection. All samples were collected from patients who provided informed consent under institutional review board-approved protocols and were stored at −80 °C until use.

### RNA sequencing and circRNA analysis

We extracted the total RNA from 3 pairs of fresh frozen BC primary tissues and liver metastatic tissues by TRIzol reagent (Invitrogen, CA, USA). Then, the RNA-seq libraries were constructed and sequenced by the Illumina HiSeq2500 platform (Illumina, San Diego, USA) according to standard protocols.

### Cell proliferation, migration and invasion assays and wound healing assays

EdU, colony formation, CCK-8, wound healing, and Transwell migration and invasion assays were performed as previously reported [17, 18].

### Chromatin immunoprecipitation (ChIP)

Formaldehyde (1%) was used for the ten-minute crosslinking reaction, which was terminated by glycine. Next, a Magnetic Bead ChIP Kit (Thermo Fisher, MA, USA) was used according to the manufacturer’s protocols. Finally, the enriched DNA was analyzed by RT-qPCR and separated by agarose gel electrophoresis.

### RNA immunoprecipitation (RIP)

The RIP assay was performed using a Magna RIP kit (Millipore, MA, USA) following the manufacturer’s protocols. The coprecipitated RNA was extracted by TRIzol (Invitrogen, CA, USA) and analyzed by RT-qPCR.

### Biotin probe synthesis and RNA pulldown assay

The biotinylated circROBO1 probe (5′-CGAAGTACTTGAACAGTCAG-3′) and control probe (5′-GAAACTGCTCGGAACGTTAA-3′) were synthesized by RiboBio (Guangzhou, China), while the pre-ROBO1 probes were transcribed using the MAXIscriptTM T7 Transcription Kit (Invitrogen, CA, USA). Then, an RNA-Protein Pull Down Kit (Thermo Fisher, MA, USA) was used according to the manufacturer’s guidelines. The abundance of hsa-miR-217-5p was detected by RT-qPCR, while the enriched protein was analyzed by western blot.

### Animal experiments

Three-week-old BALB/c female nude mice were purchased from Vital River Laboratories (Beijing, China) and housed at the Center of Experimental Animals of Sun Yat-Sen University under standard conditions. BT-549 and MCF-7 cells containing luciferase with stable circROBO1 overexpression or control cells (2x10^6^ in PBS) were subcutaneously injected into nude mice, which were sacrificed 28 days later. Before this, we intraperitoneally injected 100 µl of D-luciferin (15 µg/µl dissolved in sterilized PBS) (Yeason, Shanghai, China). After 15 minutes, images were taken with a Xenogen IVIS Lumina Series II for 1 s, and we analyzed the results with the Living Image 2.11 software package (Xenogen Corp.). The tumor volume was measured weekly and calculated according to the formula volume = width^2^/2 x length, while the tumor weight was measured after the mice were sacrificed. For the liver metastasis experiments, 1x10^6^ BT-549 cells or MCF-7 cells stably overexpressing circROBO1 with luciferase were injected into the spleens of 5- to 6-week-old female BALB/c mice; corresponding control cells were also injected into other mice. After 60 days, the mice were sacrificed, and livers were removed for further pathological assessment. The number of metastatic nodules of the liver was counted under a microscope. All the procedures were approved by the Sun Yat-Sen University Animal Care and Use Committee.

### Statistical analysis

Statistical analyses were performed with SPSS 21.0 (IBM, SPSS, IL, USA) and GraphPad Prism 9.1 (GraphPad Software Inc., CA, USA). Student’s t test was used to analyze the differences between groups, while the correlation between the groups was analyzed by the Pearson correlation coefficient. The Kaplan–Meier method was used for survival analysis, and the log-rank test was used to determine the statistical significance. * indicates *p* <0.05; ** indicates *p* <0.01; *** indicates *p* <0.001.

## Discussion

Metastasis causes the majority of cancer-related deaths worldwide [19]. Recently, circRNAs have attracted attention worldwide in the study of oncogenesis due to their unique molecular structure and tissue-/stage-specific expression, which provides a huge opportunity for using circRNAs as diagnostic markers and precise therapeutic targets for patients with cancer [20]. Therefore, it is urgent to identify novel biological functional circRNAs to elucidate how they affect BC metastasis.

In this study, we investigated the expression profile of circRNAs in primary BC tissues and matched BC-derived liver metastatic tissues by RNA-seq. Then, we focused on the circRNA circROBO1 because it was significantly upregulated in liver metastasis and associated with the poor prognosis of BC patients. Meanwhile, circROBO1 was mostly upregulated in triple negative cell lines such as MDA-MB-231 and BT-549. Next, we found that knockdown of circROBO1 could significantly inhibit cell proliferation, migration, and invasion and suppress oncogenesis and metastasis in vivo, while overexpression of circROBO1 showed the opposite effects.

FUS, an RNA-binding protein, is a member of the FET protein family and was recently reported to be a novel regulator of the biogenesis of circRNAs [14, 21]. For example, Chen et al. demonstrated that FUS could upregulate the expression of NF-IB to promote TNBC development by enhancing the biogenesis of circHIF1A [22]. Moreover, Han et al. showed that FUS could promote the biogenesis of circLONP to enhance invasion and metastasis in colorectal cancer [23]. However, the biological functions of FUS in liver metastasis of BC are still unclear. In our study, we found that FUS was upregulated in BC and could bind to the downstream intron of pre-ROBO1, the precursor of circROBO1, to promote the back splicing of pre-ROBO1 to form the mature circROBO1, as demonstrated by insertion of an HA tag into the first exon of cricROBO1 and consequent RNA pulldown assays. Therefore, higher expression of FUS might be a poor prognostic indicator in BC and might facilitate liver metastasis of BC by upregulating circROBO1.

The functions of circRNAs are correlated with their subcellular location. In our research, we showed that circROBO1 was abundant in the cytoplasm and could function by sponging miR-217-5p based on bioinformatics analysis, RNA pulldown assays, and dual-luciferase reporter assays. MiR-217-5p acts as a tumor suppressor in many cancers [24–28]. The tumor-suppressive characteristics of miR-217-5p and the interaction between circROBO1 and miR-217-5p were demonstrated in our study. Moreover, ectopic expression of miR-217-5p markedly attenuated the cancerogenic effects of circROBO1, which indicated that the functions of miR-217-5p were partially mediated by circROBO1.

KLF5 is a transcription factor that is overexpressed in the basal subtype of BC [29]. KLF5 has been reported as a potential biomarker for poor prognosis in BC [30, 31]. Moreover, KLF5 could promote cancer metastasis in a variety of cancer types [32–34]. Therefore, we identified KLF5 as a direct target gene of miR-217-5p by bioinformatic analyses and dual-luciferase assays. We found that the expression of KLF5 was positively correlated with circROBO1 and could be reversed by miR-217-5p. Our study revealed that a decrease or restoration of the expression of KLF5 could abrogate or recover the oncogenic effects of circROBO1 by miR-217-5p inhibitors or mimics, respectively. As a transcription factor, KLF5 can recognize the sequence GCCCGCCC in the promoter region. According to the JASPAR analyses, we identified the binding sites of KLF5 in the FUS promoter. ChIP and dual-luciferase assays confirmed that KLF5 could interact with the promoter of FUS and activate FUS transcription, thus leading to upregulation of circROBO1 expression. Therefore, our research revealed a positive feedback loop established by the circROBO1/KLF5/FUS axis to accelerate liver metastasis in BC.

Autophagy, a “self-eating” program, is an intracellular mechanism for protein degradation and the removal of damaged organelles via lysosomes [35, 36]. Alterations in autophagy are associated with carcinogenesis of cancers, among which BC was the first cancer genetically correlated with impairment of autophagy [37]. Beclin1 is identified as a haploinsufficient tumor suppressor that is monoallelically deleted in 50% of BC [38]. Meanwhile, low expression of BECN1 is an independent indicator of poor prognosis in BC [39]. According to a recent report that KLF5 could inhibit autophagy in prostate cancer [40], we performed RT-qPCR to find that BECN1 was negatively correlated with KLF5 in BC. As verified by ChIP and dual-luciferase assays, we confirmed that KLF5 could bind with the promoter of BECN1 and inactivate its transcription, thus leading to inhibition of autophagy, which was confirmed by IF assessment of LC3 puncta and western blott. Autophagy has multiple roles in primary tumor initiation and metastatic progression. For instance, autophagy can act as a tumor suppressor but also promote the metabolic adaptability of tumors to promote survival. Therefore, we focused on a more precise process of autophagy, which is known as “selective autophagy.” Li et al. revealed that tenascin-C is degraded by the autophagy cargo receptor p62 to induce immunosuppression mediated by autophagy deficiency in BC [41]. Moreover, Yamamoto et al. found that NBR1, a cargo receptor of autophagy, could bind with MHC-I to mediate its selective autophagy to regulate antigen presentation and anti-tumor T cell responses in pancreatic cancer [42]. All the results above showed that selective autophagy is a specific program that mediates cargo degradation for immunoregulation or carcinogenesis, which is a more accurate analysis of the influence of autophagy rather than the macroscopic scale of inhibition or activation of autophagy.

Afadin, an F-actin-binding protein, mediates cellular epithelial polarity, which is located at adherent junctions, forming a complex with actin cytoskeleton and adhesion proteins [16]. Afadin has been identified as a specific prognosis biomarker for liver metastasis in BC, and a lack of afadin was shown to markedly diminish the number of colonies in soft agar or metastatic site formation in vivo experiments [43]. The PDZ motif of afadin could interact with claudin-2 and ZO-1 to enhance tight junctions, which are essential for metastatic cells to form colonies in distant organs [44]. Moreover, afadin and nectins are necessary for the recruitment interface between cancer cells and cancer-associated fibroblasts (CAFs) [45]. In summary, afadin plays an important role in liver metastasis in BC. Next, we found that the inhibition of autophagy via inhibition of BECN1 upon overexpression of circROBO1 or KLF5 markedly increased the protein level of afadin as shown by western blot. Interestingly, the regulation of afadin occurred at the protein level but not at the mRNA level, while the mRNA level of afadin showed almost no significant change after alterations in circROBO1 or KLF5 expression. Therefore, we screened the cargo receptors of autophagy for their ability to bind afadin and found that NBR1 was the autophagic cargo receptor that bounded afadin among those tested (p62, NBR1, NDP52, TAX1BP1 and Tollip). NBR1 has been identified as a mediator of autophagy-dependent metastasis, which is an previously unrecognized autophagic substrate that potently dominates metastatic potential in preclinical models of BC [46]. Marsh et al. revealed that suppressing the accumulation of NBR1 in autophagy-deficient tumor cells markedly reversed the increase in metastatic sites in BC models [47]. We found that after inhibition of autophagy upon overexpression of circROBO1 or KLF5, the protein level of NBR1 was upregulated. Then, we found that the interaction between afadin and NBR1 was diminished when the UBA domain of NBR1 was altered, which indicated that NBR1 used its UBA domain to bind with afadin to mediate selective autophagy. Moreover, we found that EBSS-induced autophagy significantly promoted the interaction between NBR1 and afadin.

In summary, our research confirmed that the circROBO1/KLF5/FUS positive feedback loop is essential for liver metastasis in BC. We are the first to describe the crosstalk between a feedback loop and selective autophagy of afadin to provide a deeper understanding of the mechanism of circROBO1 in metastasis.

## Supplementary Information

**Additional file 1.** Supplemental Method.

**Additional file 2: Table S1.** Sequences of siRNAs used in this study. **Table S2.** Primer sequences used in RT-qPCR and PCR analysis.

**Additional file 3: Supplemental Figure S1.**

## Data Availability

The datasets used and analyzed during the current study are available from the corresponding author on reasonable request.

## Abbreviations

CircRNA: Circular RNA
BC: Breast cancer
RNA-seq: RNA sequencing
RT-qPCR: Real-time quantitative polymerase chain reaction
ceRNA: Competing endogenous RNA
miRNA: MicroRNA
TCGA: The Cancer Genome Atlas
IHC: Immunohistochemistry
IF: Immunofluorescence
FISH: Fluorescence in situ hybridization
3’UTR: 3' -untranslated region
RIP: RNA immunoprecipitation
ChIP: Chromatin Immunoprecipitation
siRNA: Small interfering RNA
