# Supplementary material for: The circROBO1/KLF5/FUS feedback loop regulates the liver metastasis of breast cancer by inhibiting the selective autophagy of afadin
Source: Mol Cancer. 2022 Jan 24;21:29. doi: 10.1186/s12943-022-01498-9 (PMC8785480; doi:10.1186/s12943-022-01498-9)
Supplement: Supplementary file 1 — Additional file 1. Supplemental Method. [file 12943_2022_1498_MOESM1_ESM.docx]

**Supplemental Method**

**siRNAs and cell transfection**

To knock down circROBO1, siRNAs which targeted the back splice junction of circROBO1 (si-circROBO1-1, si-circROBO1-2) and siRNA-NC were synthesized (GenePharma, Shanghai, China). The siRNAs of FUS, KLF5, BECN1, the mimics and inhibitors of hsa-miR-217-5p were synthesized from GenePharma (Shanghai, China) while mimics-NC and inhibitors-NC were used as controls. Transfections were conducted by Lipofectamine 3000 (Invitrogen, CA, USA) following the protocols. The sequences of siRNAs used in this research were listed in Table S1.

**Fluorescence in situ hybridization (FISH)**

Cy3-lableled circROBO1 (5’-AGTCATCCCGAAGTACTTGAACAGTCAGAG-3’) probe (GenePharm, Shanghai, China) was used to localize the circROBO1 in BC cells and tissues. FISH assays were performed in cell and tissues using the Fluorescent In Situ Hybridization Kit (GenePharm, Shanghai, China) following the protocols. The images of the results were taken by the Olympus BX53 fluorescence microscope (Olympus, Japan).

**Western blot analysis**

Concisely, the proteins were extracted and isolated by 6-12% SDS-PAGE and the protein were transferred to the PVDF membranes (Millipore, MA, USA). Next, we used 5% skim milk powder to block the membranes and incubated the membranes with primary antibodies over night at 4°C. After that, secondary antibodies were incubated with the membrane for 1h. Eventually, the chemiluminescence was executed to detect the protein bands. The primary antibodies that we used in this study: anti-KLF5 (21017-1-AP, Proteintech, China), anti-FUS (11570-1-AP, Proteintech, China), anti-mTOR (66888-1-Ig, Proteintech, China), anti-p-mTOR(67778-1-Ig, Proteintech, China), anti-AKT (#4691, CST, USA), anti-p-AKT (#4060, CST, USA), anti-PI3K(20584-1-AP, Proteintech, China), anti-NBR1(16004-1-AP, Proteintech, China), anti-AFDN (#13531, CST, USA), anti-BECN-1 (#3495, CST, USA), anti-ATG5 (#12994, CST, USA), anti-ATG16L1 (#8089, CST, USA), anti-LC3-I/II (#12741, CST, USA), anti-p62 (#88588, CST, USA), anti-GAPDH (60004-1-Ig, Proteintech, China), anti-HA Mouse antibody (66006-2-Ig, Proteintech, China), anti-Flag Mouse antibody(66008-3-Ig, Proteintech, China).The secondary antibodies that we used in this study: anti-Rabbit-IgG HRP-link antibody (#7074, CST, USA), anti-Mouse-IgG HRP-link antibody (#7076, CST, USA).

**Plasmid construction**

To construct circROBO1 overexpression vector, we cloned the full length of human circROBO1 into the PLCDH-ciR vector (Geenseed Biotech, Guangzhou, China) which included the front and back circular frame while the mock vector that only contained a stuff was used as control. To establish stably transfected BC cells, firstly we transfected transiently circROBO1 or vector with two assistant vectors into HEK293T cells to produce lentivirus. After that, we infected the target cells with lentivirus produced above and screened stably transfected cells with puromycin. To construct FUS and KLF5 overexpression vectors, we cloned the length of human FUS and KLF5 into pcDNA3.1(+) (Thermofisher, MA, USA). Moreover, we cloned the promotor of FUS and BECN1 into pEZX-FR01 plasmid (GeneCopeia, Guangzhou, China). All the vectors above were verified by sequencing. For pc-HA-ROBO1 vector, we cloned 2000bp upstream of ROBO1 exon5 and 2000bp downstream of ROBO1 exon 8 and inserted a HA tag into the 5’ side of exon5. After that we integrated sequences mentioned above into pcDNA3.1(+) to construct pc-HA-ROBO1.

**Immunofluorescence (IF) and immunohistochemistry (IHC)**

IF and IHC assays were conducted as previously reported [19]. For IF experiment, tissues or cells were incubated with primary antibodies against KLF5 (1:50 dilution) (21017-1-AP, Proteintech, China) at 4°C overnight, then incubated with fluorescein CoraLite594–conjugated secondary antibodies (SA00013-4, Proteintech, China) and imaged by using Olympus fluorescence microscope (Olympus, Japan). For IHC assays, paraffin sections were incubated with the anti-KLF5 primary antibody against (1:100 dilution) (21017-1-AP, Proteintech, China). Images were photographed by Nikon Eclipse 80i microscope (Nikon, Tokyo, Japan).

**RNase R, actinomycin D assays, and nuclear-cytoplasmic fractionation**

Rnase R assays were performed at 37°C with 4 U/μg of RNase R (Geenseed Biotech, Guangzhou, China) for 30min. Additionally, the RNA of BC cells was extracted after treatment with 120 ng/ml actinomycin D (Solarbio, Shanghai, China) for 4h, 8h, 12h and 24h respectively. Nuclear and cytoplasmic RNA from BC cells were separated by the PARIS Kit (Invitrogen, CA, USA) according to the protocols.

**Dual-luciferase reporter assay**

The sequences of circROBO1 or KLF5 3’UTR that contained the wild type (WT) or mutant type (MT) binding sites of hsa-miR-217-5p and the promotors of FUS and BECN1 that contained the wild type (WT) or mutant type (MT) binding sites of KLF5 were all synthesized by Ruibiotech (Beijing, China). Then we cloned the WT or MT sequences of circROBO1 or KLF5 3’UTR into pmirGLO (Promega, USA). After that, co-transfections were conducted with the corresponding plasmids and mimics/mimics-NC or inhibitors/inhibitor-NC of hsa-miR-217-5p in 293T cells respectively. Meanwhile, the WT or MT sequences of promotors of FUS or BECN1 were cloned into pEZX-FR01 (GeneCopoeia, MD, USA). Next, we performed co-transfection with plasmids constructed above with KLF5-siRNA or KLF5-overexpression plasmid in 293T cells as well. The activities of Renilla and Firefly luciferase of all co-transfections above were analyzed by Dual Luciferase Reporter Assay Kit (Promega, WI, USA) after 48h of incubation while Renilla was used as internal reference.

**Chromatin immunoprecipitation (ChIP)**

1% formaldehyde was used for the ten-minute crosslinking reaction which was terminated by glycine. Next, Magnetic Bead ChIP Kit (Thermofisher, MA, USA) were used for the following procedure according to the manufacture’s protocols. Briefly, the DNA-protein complex was digested by MNase and sonicated to produce 200-800bp DNA fragments. After incubation with anti-KLF5 antibody (21017-1-AP, Proteintech, China) or IgG negative control antibody (30000-0-AP, Proteintech, China) overnight, ChIP Grade Protein A/G Magnetic Beads were used for immunoprecipitation of DNA-protein complex in lysates. Finally, the enriched DNA was analyzed by RT-qPCR and nuclear acid electrophoresis. And the sequences of primers were listed in Additional file.

**RNA immunoprecipitation (RIP)**

RIP assay was performed by Magna RIP kit (Millipore, MA, USA) following to the protocols. Concisely, the Magnetic Beads Protein A/G were incubated with anti-FUS antibody (11570-1-AP, Proteintech, China) or IgG negative control antibody (30000-0-AP, Proteintech, China) respectively. Then the lysates of cells were incubated was antibody-coated magnetic beads overnight. Finally, the coprecipitated RNA was extracted by TRIzol (Invitrogen, CA, USA) and analyzed by RT-qPCR.

**Biotin probes synthesis and RNA pull down assay**

The biotinylated circROBO1 probe (5’-CGAAGTACTTGAACAGTCAG-3’) and control probe (5’-GAAACTGCTCGGAACGTTAA-3’) were synthesized by Ribobio (Guangzhou, China) while the probes of pre-ROBO1 were transcribed using MAXIscriptTM T7 Transcription Kit (Invitrogen, CA, USA). Then RNA-Protein Pull Down Kit (Thermofisher, MA, USA) was used to perform the following procedure according to the manufacture’s guidelines. Briefly, prepared biotinylated probe was incubated with streptavidin magnetic beads around half an hour to produce magnetic beads-probe complex. After that, magnetic beads-probe complex was incubated with cell lysates overnight at 4°C and the resultants were washed and eluted for RNA analysis by TRIzol (Invitrogen, CA, USA) or protein analysis by SDS-PAGE loading buffer (Beyotime, Shanghai, China). The abundance of hsa-miR-217-5p was detected by RT-qPCR while the enriched protein was analyzed by western blot.
