# Supplementary material for: The circROBO1/KLF5/FUS feedback loop regulates the liver metastasis of breast cancer by inhibiting the selective autophagy of afadin
Source: Mol Cancer. 2022 Jan 24;21:29. doi: 10.1186/s12943-022-01498-9 (PMC8785480; doi:10.1186/s12943-022-01498-9)
Supplement: Supplementary file 2 — Additional file 2: Table S1. Sequences of siRNAs used in this study. Table S2. Primer sequences used in RT-qPCR and PCR analysis. [file 12943_2022_1498_MOESM2_ESM.docx]

**Table S1 Sequences of siRNAs used in this study**

| **Definition** | **sequences** |
| --- | --- |
| si-circROBO1-1 | 5'-GAAAACACAAGAUAUGAAA-3' |
| si-circROBO1-2 | 5'-CACAAGAUAUGAAAUCCGA-3' |
| si-NC | 5'-UUCUCCGAACGUGUCACGU-3' |
| si-FUS | 5'-CAGAGUUACAGUGGUUAUA-3' |
| si-KLF5 | 5’-GCAGACUGCAGUGAAACAA-3’ |
| si-BECN1 | 5’-CUGGACACGAGUUUCAAGA-3’ |
| si-NBR1 | 5’-GCGCUUAAGAUGGCAGUUA-3’ |

**Table S2 Primer sequences used in RT-qPCR and PCR analysis**

| **Gene** | **Primer sequences** |
| --- | --- |
| GAPDH | F: 5'-GAAGGTGAAGGTCGGAGTC-3' |
|  | R: 5'-GAAGATGGTGATGGGATTTC-3' |
| U6 | F: 5'-CGAGCACAGAATCGCTTCA-3' |
|  | R: 5'-CTCGCTTCGGCAGCACATAT-3' |
| circROBO1 | F: 5'-GCTGGTGACATGGGTTCATACA-3' |
|  | R: 5'-AAATGGTGGGCTCAGGATGG-3' |
| ROBO1 | F: 5'-GGGACCCTATTTCCACTCCC-3' |
|  | R: 5'-GGGAGCCTGAACAGAGACAT-3' |
| FUS | F: 5'-CAAGGCCTGGGTGAGAATGT-3' |
|  | R: 5'-TTGCCTCTCCCTTCAGCTTG-3' |
| KLF5 | F: 5'-CCTGGTCCAGACAAGATGTGA-3' |
|  | R: 5'-GAACTGGTCTACGACTGAGGC-3' |
| ATG12 | F: 5'-CTGCTGGCGACACCAAGAAA-3' |
|  | R: 5'-CGTGTTCGCTCTACTGCCC-3' |
| BECN1 | F: 5'-GGTGTCTCTCGCAGATTCATC-3' |
|  | R: 5'-TCAGTCTTCGGCTGAGGTTCT-3' |
| NBR1 | F: 5'-AGATGGCAGTTAAACAGGGAAAC-3' |
|  | R: 5'-GTGGGGCTTCATCAACGACA-3' |
| ATG7 | F: 5'-CAGTTTGCCCCTTTTAGTAGTGC-3' |
|  | R: 5'-CCAGCCGATACTCGTTCAGC-3' |
| ULK1 | F: 5'-AGCACGATTTGGAGGTCGC-3' |
|  | R: 5'-GCCACGATGTTTTCATGTTTCA-3' |
| Afadin  FUS E1  FUS E2  FUS E3  BECN1 E1  BECN1 E2  pc-HA-ROBO1 | F: 5'-ATTTCGACCTGATATGCGAATGC-3' |
|  | R: 5'-CAAATCTGCCTTCCCGATCAT-3'  F: 5'-CTGTGACTCCAGTTTCGTCC-3'  R: 5'-CTCTCTCCTCGGCAGGACTA-3'  F: 5'-GAGGTCAACCCTCTCTGGTC-3'  R: 5'-GACCTGAAAGACAGCAGGGATT-3'  F: 5'-AAGTCAACCTCAGGGCGCAG-3'  R: 5'-CTTTCACCCAGGATGGCGAG-3'  F: 5'-CAAGCGATTCTCCTGCCTCA-3'  R: 5'-TTCGAGACCAGCCTGGCCAA-3'  F: 5'-CAGGTAAGAATTCCTTCCTCA-3'  R: 5'-TTCGAGACCAGCCTGGCCAA-3'  F: 5'-GTCAGGCACGTCGTAAGGATA-3'  R: 5'-AAATGGTGGGCTCAGGATGG-3' |
