# Supplementary material for: The circROBO1/KLF5/FUS feedback loop regulates the liver metastasis of breast cancer by inhibiting the selective autophagy of afadin
Source: Mol Cancer. 2022 Jan 24;21:29. doi: 10.1186/s12943-022-01498-9 (PMC8785480; doi:10.1186/s12943-022-01498-9)
Supplement: Supplementary file 3 — Additional file 3: Supplemental Figure S1. [file 12943_2022_1498_MOESM3_ESM.pdf]

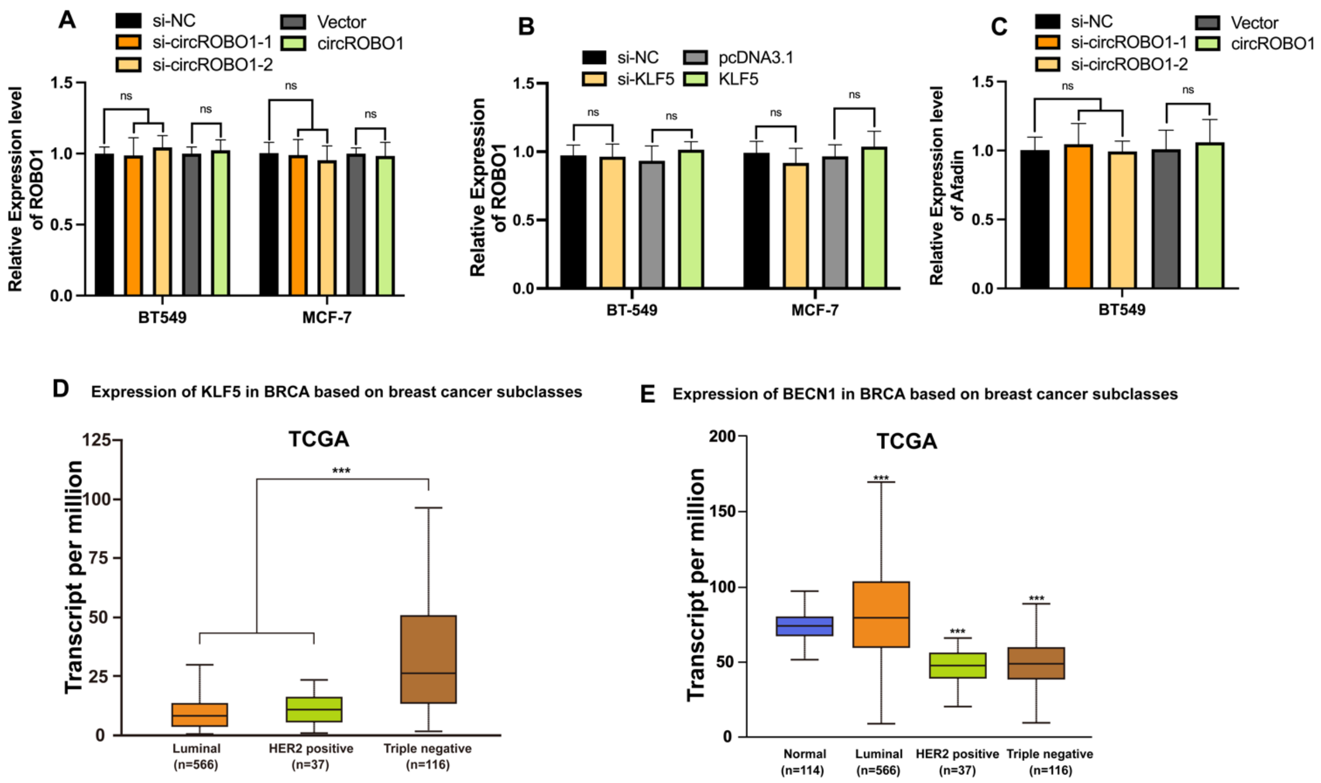

A. RT-qPCR was performed to detect no significance change of ROBO1 after transfected with siRNAs of circROBO1 or overexpression vector of circROBO1.

B. B RT-qPCR was conducted to identify the change of ROBO1 after knockdown or overexpression of KLF5

C. C The mRNA expression of afadin was validated by RT-qPCR after knockdown or overexpression of circROBO1

D. D The expression of KLF5 was upregulated in triple negative subtype of BC compared to luminal or HER2 positive subtypes according to TCGA.

E. The expression of BECN1 was identified to downregulated in HER2 positive and triple negative subtypes of BC compared to normal tissues according to TCGA.
